# Extracellular anti-angiogenic proteins augment an endosomal protein trafficking pathway to reach mitochondria and execute apoptosis in HUVECs

**DOI:** 10.1038/s41418-018-0092-9

**Published:** 2018-03-09

**Authors:** Mo Chen, Tao Qiu, Jiajie Wu, Yang Yang, Graham D. Wright, Min Wu, Ruowen Ge

**Affiliations:** 1 Department of Biological Sciences, Faculty of Science, National University of Singapore, Singapore, 117543 Singapore; 2 Institute of Medical Biology, Agency for Science, Technology & Research (A*STAR), Singapore, 138648 Singapore

## Abstract

Classic endocytosis destinations include the recycling endosome returning to the plasma membrane or the late endosome (LE) merging with lysosomes for cargo degradation. However, the anti-angiogenic proteins angiostatin and isthmin, are endocytosed and trafficked to mitochondria (Mito) to execute apoptosis of endothelial cells. How these extracellular proteins reach mitochondria remains a mystery. Through confocal and super-resolution fluorescent microscopy, we demonstrate that angiostatin and isthmin are trafficked to mitochondria through the interaction between LE and Mito. Using purified organelles, the LE–Mito interaction is confirmed through in vitro lipid-fusion assay, as well as single vesicle total internal reflection fluorescent microscopy. LE–Mito interaction enables the transfer of not only lipids but also proteins from LE to Mito. Angiostatin and isthmin augment this endosomal protein trafficking pathway and make use of it to reach mitochondria to execute apoptosis. Cell fractionation and biochemical analysis identified that the cytosolic scaffold protein Na+/H+ exchanger regulatory factor 1 (NHERF1) associated with LE and the t-SNARE protein synaptosome-associated protein 25 kDa (SNAP25) associated with Mito form an interaction complex to facilitate LE–Mito interaction. Proximity ligation assay coupled with fluorescent microscopy showed that both NHERF1 and SNAP25 are located at the contacting face between LE and Mito. RNAi knockdown of either NHERF1 or SNAP25 suppressed not only the mitochondrial trafficking of angiostatin and isthmin but also their anti-angiogenic and pro-apoptotic functions. Hence, this study reveals a previously unrealized endosomal protein trafficking pathway from LE to Mito that allows extracellular proteins to reach mitochondria and execute apoptosis.

## Introduction

Mitochondria (Mito) are not only the central power stations in the cell, they also serve as hubs for various metabolic and signaling processes [1]. Extracellular anti-angiogenic proteins such as angiostatin (AS) and isthmin (ISM) [2–4], are endocytosed into endothelial cells and subsequently trafficked to Mito to execute apoptosis. The *H. pylori* VacA toxin is also endocytosed into endosomes and then transferred to Mito to induce apoptosis [5, 6]. On the other hand, cell surface receptors such as epidermal growth factor receptor (EGFR) [7–9] and glucose-regulated protein 78 kDa (GRP78) [3], have also been reported to be translocated to Mito. Furthermore, certain conventional mitochondrial proteins, such as ATP synthase [10, 11] and voltage-dependent anion-selective channel (VDAC) [12], also reside on the cell surface and serve as receptors. These findings suggest the existence of a protein translocation mechanism between extracellular environment and cell surface to Mito.

The classic endocytic pathway starts with vesicle budding from the plasma membrane (PM) to form an early endosome (EE), which later either recycles back to the PM or matures into a late endosome (LE) before fusion with a lysosome for cargo degradation [13]. Recently, endocytic routes from the extracellular environment/cell surface to nucleus or Golgi apparatus have also been reported [14, 15]. As Mito is not a known destination of endocytosis, we hypothesized that these extracellular anti-angiogenic proteins may have first escaped from endosomes and subsequently entered Mito from the cytosol. Surprisingly, our findings show that both mitochondrial targeting and apoptosis-inducing function of AS and ISM require intact LE. Using multiple experimental approaches, we demonstrate that Mito is a previously unrealized endocytosis destination. AS and ISM reach Mito via direct LE–Mito interaction and/or fusion.

## Results

### Intact LEs are required for AS and ISM trafficking to Mito

**Fig. 1:**
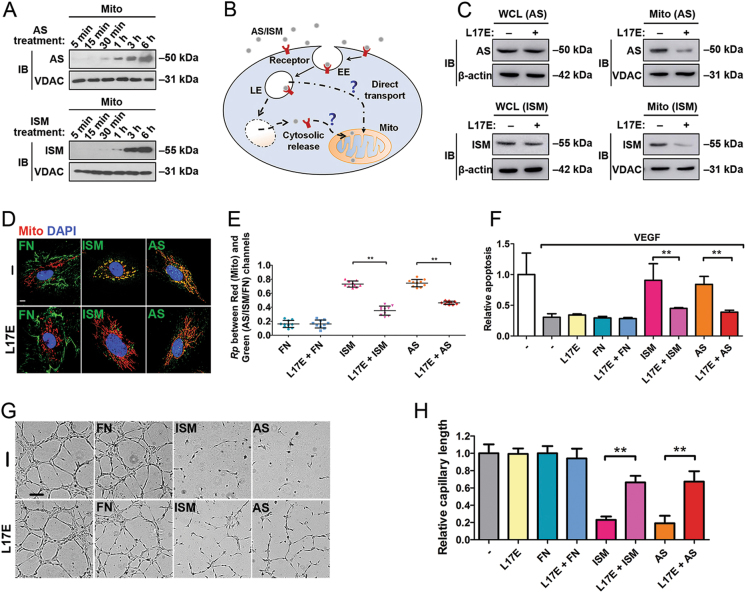
Intact LEs are required for AS/ISM trafficking to mitochondria. **a** WB shows AS/ISM are trafficked to mitochondria in a time-dependent manner. **b** The working hypothesis of the endocytic trafficking pathways. **c** L17E endosomolytic peptide reduced the mitochondrial targeting of AS/ISM without affecting the total endocytosis of AS and ISM by WB. **d**, **e** The mitochondrial targeting of AS and ISM was blocked by L17E. HUVECs treated by AS/ISM/FN with or without L17E were labeled by MitoTracker (Red). The colocalization between AS/ISM/FN and mitochondria was determined by Pearson’s correlation coefficient. **P < 0.01, *n* = 10. Error bars denote SD. Scale bar, 5 μm. **f** The pro-apoptotic function of AS and ISM was suppressed by L17E peptide in the apoptosis assay. FN, which does not induce EC apoptosis, served as a protein control. **P < 0.01, *n* = 3. Error bars denote SD. **g**, **h** The anti-angiogenic effect of AS and ISM was reduced by L17E in the tube formation assay. Tube formation of HUVECs on Matrigel was inhibited by AS and ISM and the inhibition was reduced by L17E treatment. FN, which does not inhibit angiogenesis, served as a protein control. The total tube length was quantified by ImageJ. **P < 0.01, *n* = 3. Error bars denote SD. Scale bar, 200 μm

Treatments of human umbilical vein endothelial cells (HUVECs) with recombinant AS and ISM lead to the internalization and accumulation of both proteins in Mito in a time-dependent manner, whereas the extracellular fibronectin (FN) remains extracellular (Fig. 1a). Fluorescent staining confirmed that both AS and ISM become colocalized with Mito, whereas FN remains in the extracellular matrix (Fig. S1). As Mito is not a known destination of endocytosis, we reasoned that AS and ISM could possibly first escape from endosomes to cytosol after endocytosis and subsequently enter Mito from the cytosol. To explore this possibility, we treated HUVECs with the pH-sensitive endosomolytic peptide L17E [16, 17]. This peptide is endocytosed into endosomes and specifically disrupts the LE membrane in the acidic pH environment to release endocytosed proteins from LE into the cytosol. If the endocytosed AS and ISM are trafficked to Mito from the cytosol after being first released from the endosomal system, L17E treatment should enhance the mitochondrial trafficking of these two proteins (Fig. 1b). Surprisingly, treatment with L17E significantly blocked the mitochondrial trafficking of both AS and ISM without affecting their endocytosis (Figs. 1c-e). As mitochondrial trafficking of AS and ISM is important for their anti-angiogenic and pro-apoptotic functions [2, 3], we further verified that L17E treatment indeed significantly reduced the pro-apoptotic and anti-angiogenic functions of both proteins (Figs. 1f–h). These results show that intact LE is not only critical for extracellular AS and ISM to be trafficked into Mito, it is also critical for their anti-angiogenic and pro-apoptotic functions. It seems that AS and ISM make use of rather than escape from the endosomal system to reach Mito.

### LE but not EE contacts Mito

Previous reports have demonstrated that iron can be transferred directly from endosome to Mito, likely through endosome–Mito interactions [18–20]. However, it is not clear whether it is EE or LE that contacts Mito for iron transfer and whether other materials can also be transferred. Our findings above prompted us to examine if AS and ISM could be transferred from endosomes to Mito through direct contact of organelles.

**Fig. 2:**
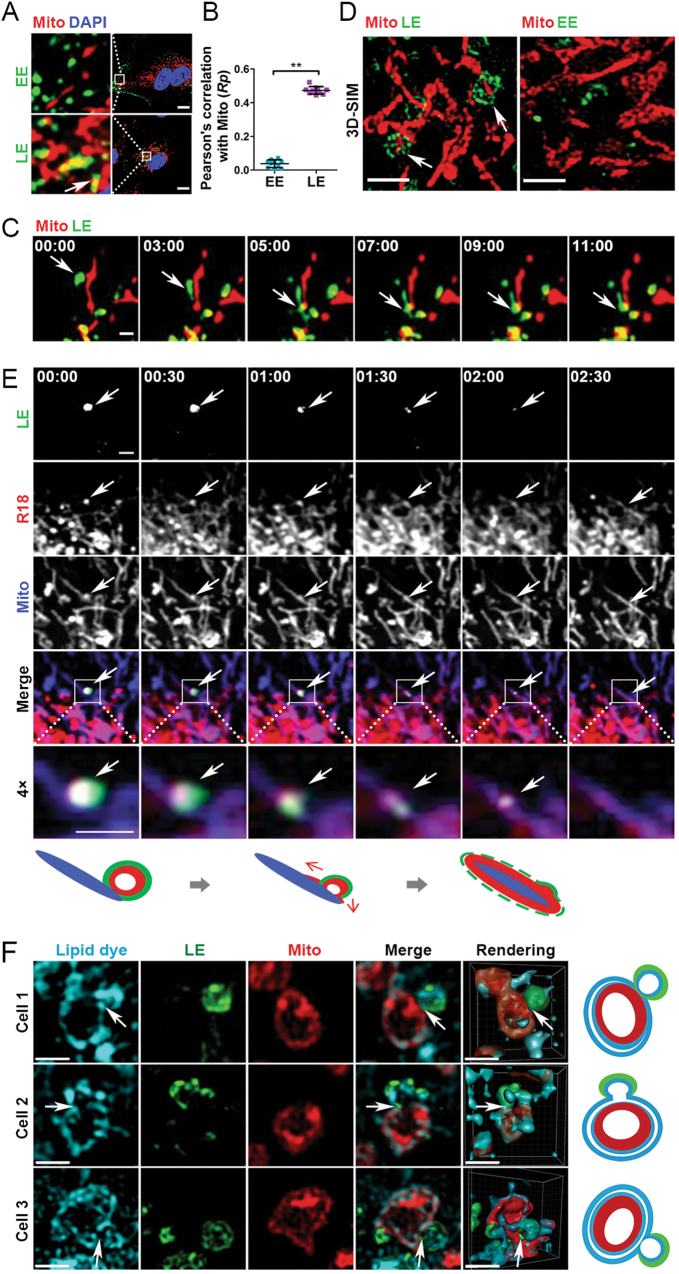
LE directly interacts with mitochondria and results in membrane merging and hybrid structure formation. **a** Confocal imaging revealed that LE and Mito show more overlap than EE and Mito. HUVECs were labeled by the EE/LE marker (Rab5-GFP/Rab7-GFP) and MitoTracker (red). The nuclei were counterstained with DAPI (blue). Scale bar: 5 μm. **b** Quantification of the Pearson’s correlation coefficient between the EE/LE and Mito. LE–Mito presents significantly higher Rp than EE-Mito, indicating colocalization of LE and Mito. **P < 0.01, *n* = 10. Error bars denote SD. **c** Time-lapse confocal imaging of the direct interaction between LE and Mito. Scale bar, 1 μm. **d** Super-resolution microscopy (3D-SIM) shows a direct interaction between LE and Mito. HUVECs were imaged by 3D-SIM and conventional wide-field fluorescence microscopy. Each frame is a maximum-intensity z-projection (MIP) over a 0.7 μm range containing the branchial mitochondria and vesicular endosome. Scale bar, 1 μm. **e** Time-lapse live-cell confocal imaging of R18 lipid dye labeled LE merging into Mito. A 4× expanded image of the merging LE–Mito (within the white box) is also shown and a schematic depiction of the corresponding image is presented at the bottom. Scale bar, 1 μm. HUVECs were labeled by the LE marker (Rab7-GFP), R18 lipid dye (Red, labels PM) and MitoTracker (Far-red). **f** 3D-SIM shows the interacting LEs and Mito and the formation of LE–Mito hybrid structure in HUVECs. HUVECs were labeled by the LE marker (Rab7-GFP), Maroon far-red lipid dye (pseudocolor Cyan) and MitoTracker (red). Each frame is a MIP over a 125-375 nm z-range to show the optimal membrane structure of LE and mitochondria. The 3D-rendering (rendering row) of the image and a schematic drawing of the interacting LE–Mito are presented at the bottom. Scale bar, 1 μm

We first investigated the colocalization of EE and LE with Mito in HUVECs. EE and LE are labeled by Rab5-GFP (Ras-related in brain 5-green fluorescent protein) and Rab7-GFP, respectively, through recombinant baculovirus infection. Mito are labeled by MitoTracker, a fluorescent dye that covalently binds mitochondrial matrix proteins located at the mitochondrial inner membrane [21–23]. Mito in HUVECs are present in both branch and rod-shaped forms with the majority of them in a compact form localized in the perinuclear region (Fig. 2a). Similar to other cells, EE and LE show distinct distribution patterns in HUVECs, with EE localizing in the cell periphery and LE residing in the perinuclear region (Fig. S2A). Using Pearson’s correlation coefficient to quantify the colocalization between endosomes and Mito, we observed a significantly higher association between LE and Mito compared with EE and Mito (Figs. 2a, b). Live-cell confocal microscopy confirmed that LE directly contacts Mito (Fig. 2c and Video 1). Three-dimensional (3D) structured illumination super-resolution microscopy (3D-SIM) further confirmed that vesicular LE, but not EE, contacts branched Mito (Fig. 2d).

### LE interacts with and merges into Mito

Under differential interference contrast (DIC) microscopy, the subcellular structures of vesicular LE and branched Mito in HUVECs were evident (Fig. S2B). Some LE and Mito merged into one structure (Fig. S2C, panels 1–3), whereas others are present at juxtaposition (Fig. S2C, panel 4). If LEs merge with Mito structurally, lipid transfer/mixing would occur. Using R18, a membrane-impermeable lipophilic fluorescent dye to label the PM of HUVECs, it was observed that R18 fluorescent signal translocated into Mito in 3 h (Fig. S2, D & F). Similarly, labeling HUVECs membrane with another membrane-impermeable lipid dye, PKH26, also led to the enrichment of the dye in Mito (Fig. S2, E & G).

To capture the lipid transfer from LE to Mito, HUVECs were immediately imaged after labeling the PM by the two lipid dyes. As expected, all LEs carried the lipid dye due to their PM origin. The LE marker Rab7-GFP was initially restricted within the LE vesicular structures. However, lipid dyes originated from the PM and present on LE subsequently migrated into tubular Mito. Time-lapse live-cell imaging demonstrated the gradual transfer of R18-labeled lipid from LE to Mito (Fig. 2e, arrow, panels from left to right and Video 2). LE first contacts Mito, where R18-labeled lipid is only located in LE (vesicle shape). Subsequently, the R18 lipid in LE gradually spread and merged into the contacting mitochondrial branch, eventually become invisible due to its dilutions into the much larger Mito. These observations demonstrate that lipid components from the PM can be transferred into Mito through LE–Mito contact and merging.

MitoTracker labels mitochondrial matrix proteins localized on the luminal side of the inner membrane, presenting a hollow matrix center under 3D-SIM (Fig. 2f) [23]. The external surface of some LEs (Rab7-GFP^+^) are in contact with the mitochondrial matrix (MitoTracker red), suggesting fused hybrid structure (Fig. 2f). In addition, some LEs were observed to contact Mito and then quickly dissociate from Mito (Video 3). Hence, the kiss-and-run type of LE–Mito interaction is also likely to occur in the endothelial cells. These results show that LE and Mito interact with each other with some LEs merging into Mito.

### AS and ISM utilizes and enhances the mitochondrial trafficking pathway

**Fig. 3:**
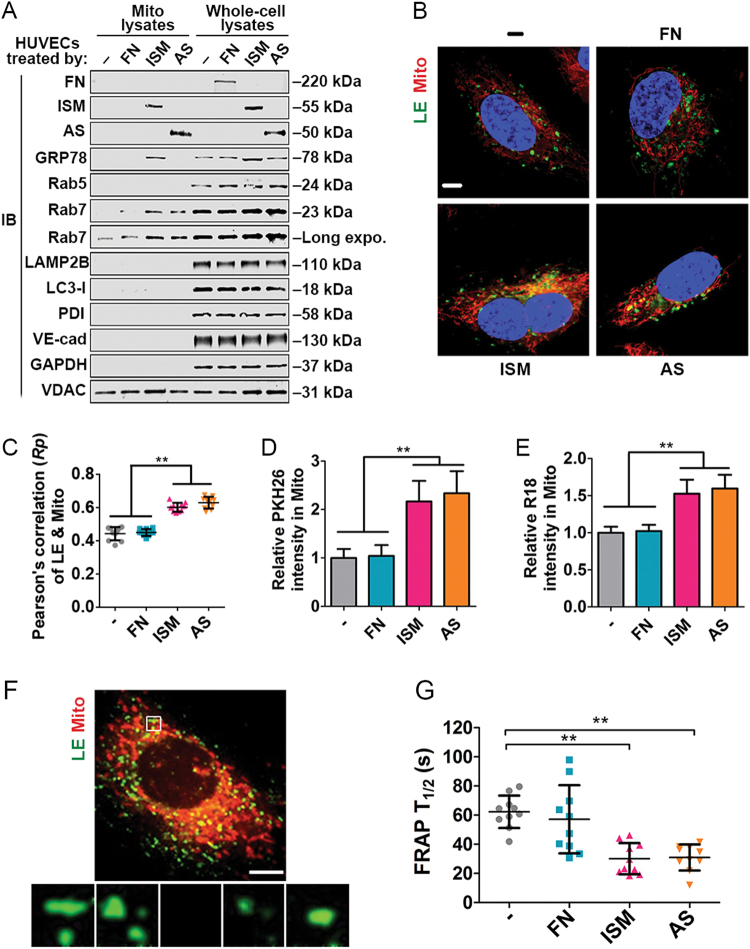
AS and ISM are trafficked to mitochondria and enhance LE–Mito interaction. **a** AS and ISM treatment enhanced the presence of Rab7 in isolated mitochondria shown by WB. The mitochondrial fraction of AS, ISM, and FN-treated HUVECs was isolated and immunoblotted for the LE marker (Rab7), EE marker (Rab5), lysosome marker (LAMP2B), autophargosome marker (LC3B), ER marker (PDI), plasma membrane marker (VE-cadherin), cytosol marker (GAPDH), mitochondrial marker (VDAC), and ISM and ISM’s cell surface receptor (GRP78). A longer exposure of Rab7 WB is also included to show the presence of this protein in Mito under natural condition. **b, c** AS and ISM but not FN increased the association between LE and Mito as shown by confocal imaging. HUVECs labeled by the LE marker (Rab7-GFP) and MitoTracker (Red) were treated with AS, ISM, and FN. Colocalization between LE and Mito was determined by Pearson’s correlation coefficient. **P < 0.01, *n* = 10. Scale bar, 5 μm. **d**, **e** AS and ISM enhanced the transfer of PKH26/R18-labeled lipid from the PM to mitochondria. FN treatment serves as a control. HUVECs labeled by PKH26 and R18 lipid dye were treated with AS, ISM and FN. Mitochondrial fraction was isolated and fluorescent intensity of the lipid dyes measured. **P < 0.01, *n* = 3. Error bars denote SD. **f**, **g** AS and ISM significantly boosted the dynamics of LE targeted to Mito area shown by FRAP. Obviously shortened T_1/2_ for fluorescence recovery of bleached LE (green) in Mito (red) area were observed upon ISM and AS treatment. HUVECs labeled by the LE marker (Rab7-GFP) and MitoTracker (Red) were treated with AS/ISM/FN and subjected to FRAP analysis. **P < 0.01, *n* = 10. Error bars denote SD. Scale bar, 5 μm

Notably, treatment with AS and ISM, but not FN, enhanced LE–Mito interaction and colocalization (Figs. 3a-c). Western Blot (WB) using protein lysates from isolated Mito demonstrated that treatment of AS and ISM lead to an increase of LE–Mito association, with more Rab7 protein present in the mitochondrial fraction. In comparison, markers for other organelles such as EE (Rab5), lysosome (LAMP2B), autophagosome (LC3-II), ER (PDI), PM (VE-Cad) and cytosol (GAPDH) were not increased in the same mitochondrial fraction. AS and ISM also enhanced lipid transfer from PM into Mito, with significantly enhanced PM labeling dye PKH26 and R18 in Mito under AS and ISM treatment (Figs. 3d, e). In addition, the dynamics of Mito-targeting LEs (green) is significantly increased when examined by fluorescence recovery after photo-bleaching (FRAP). The green fluorescence of LE that colocalized with Mito (red) was selectively photo-bleached. FRAP was observed within a few minutes, with the T_1/2_ of green fluorescence recovery significantly shortened by treatment with AS or ISM (Figs. 3f, g). These results show that extracellular AS and ISM can augment LE–Mito interaction to facilitate their trafficking from the extracellular environment to Mito through endocytosis.

Consistently, both confocal fluorescent imaging (Fig. S3A) and fluorescent quantification via fluorimeter measurement of isolated Mito (Fig. S3B) indicated that suppressing clathrin-mediated endocytosis by chlorpromazine treatment reduced the amount of R18 or PKH26 transferred from PM to Mito. This result suggests that endocytosis contributes to lipid transfer from PM to Mito, further support the existence of an intracellular trafficking pathway via direct LE–Mito interaction following endocytosis.

### LE interacts with Mito in vitro, leading to lipid exchange and mixing

**Fig. 4:**
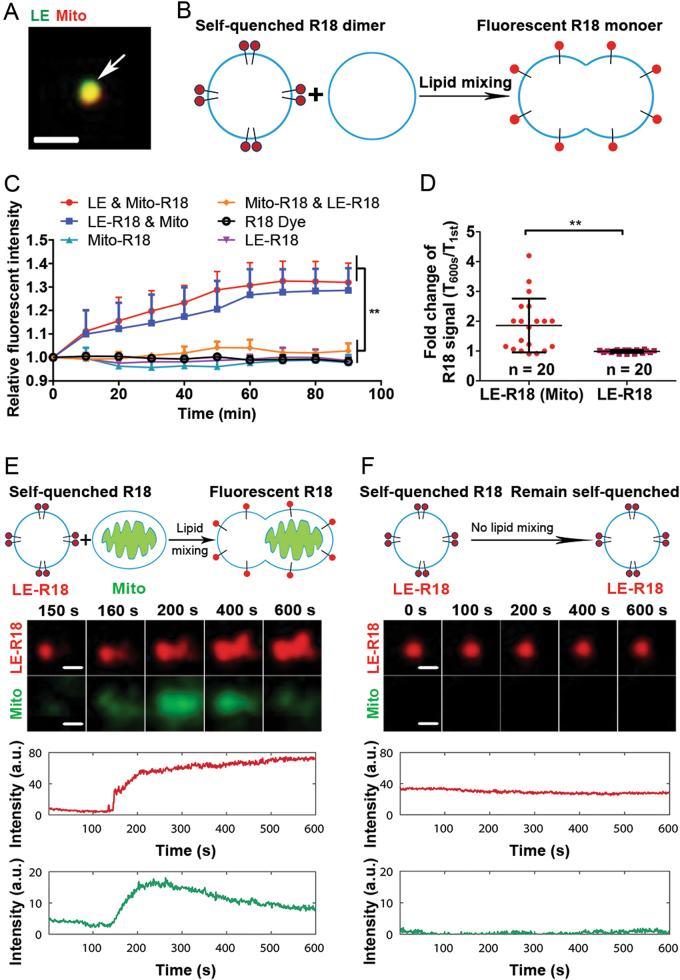
LEs directly interact with Mito in vitro. **a** LE interacts with Mito in vitro determined by confocal microscopy. Purified LE (green, Rab7-GFP labeled) and Mito (labeled by MitoTracker, red) were mixed in PBS in a Petri dish. Their direct interaction was examined by confocal microscopy. Scale bar, 1 μm. **b**, **c** In vitro lipid-mixing assay shows that LEs directly interact with Mito. By mixing either isolated R18-labeled mitochondria with unlabeled LE or vice versa can lead to de-quenching and increase of R18 fluorescence, a phenotype indicating direct membrane interaction and lipid mixing. No fluorescence increase can be observed in the control groups with R18 dye alone, R18-labeled LEs alone, R18-labeled Mito alone, or the mixture of both R18-labeled LE and R18-labeled mitochondria overtime. **P < 0.01, *n* = 3. **d** Quantification of R18 fluorescence intensity changes of 20 individual R18-LE interacting with Mito and R18-LE alone by total internal reflection fluorescent microscopy (TIRFM). **P < 0.01. **e** TIRFM determination of the fluorescent intensity of a single R18-labeled LE gradually increased upon interaction with a single Mito in vitro. Images on top are from selective time points to demonstrate the R18 fluorescence increased upon the interaction of R18-labeled LE and unlabeled Mito. Scale bar, 1 μm. The graph showed R18 fluorescence (red) de-quenched overtime when an R18‐labeled LE encountered an unlabeled Mito (positive in MitoTracker Green) until the end of recording. The MitoTracker green was gradually bleached over time. **f** The fluorescent intensity of R18-labeled LE remained unchanged on its own by TIRFM. Scale bar, 1 μm

To directly visualize and study LE–Mito interaction, we purified fluorescent-labeled LE and Mito, respectively, and studied them in vitro using a classic lipid-mixing assay. As shown by confocal imaging, LE directly interacts Mito when mixed in vitro (Fig. 4a). R18 dye in lipid membrane is self-quenched at high concentrations [24]. Mixing with unlabeled membrane will result in dilution and de-quenching of this dye, leading to an increase of fluorescent signal (Fig. 4b). Indeed, mixing either R18-labeled LE (R18-LE) with unlabeled Mito or R18-labeled Mito (Mito-R18) with unlabeled LE led to a time-dependent increase in fluorescent signal (Fig. 4c). Consistently, using total internal reflection fluorescence microscopy (TIRFM), the increase of R18 fluorescent signal of a single vesicle was observed when R18-labeled LE encountered a MitoTracker green-labeled Mito. In comparison, the R18 signal remained unchanged in the R18-labeled LEs alone sample (Figs. 4d-f). These results further support that direct membrane interaction and lipid mixing occurred upon LE–Mito interaction.

### The cytosolic scaffold protein NHERF1 is associated with LE and facilitates LE–Mito interaction

To study how LEs interact with Mito, we determined whether the interaction depends on surface molecules on these organelles and whether it is calcium dependent. In vitro lipid-mixing assay revealed that the lipid mixing between LE and Mito is abolished by prior high-salt wash of either LE or Mito, but is not affected by adding EGTA or Ca^2+^ (Fig. S4). These results suggest that surface-associated molecules mediate the LE–Mito interaction in a calcium-independent manner.

**Fig. 5:**
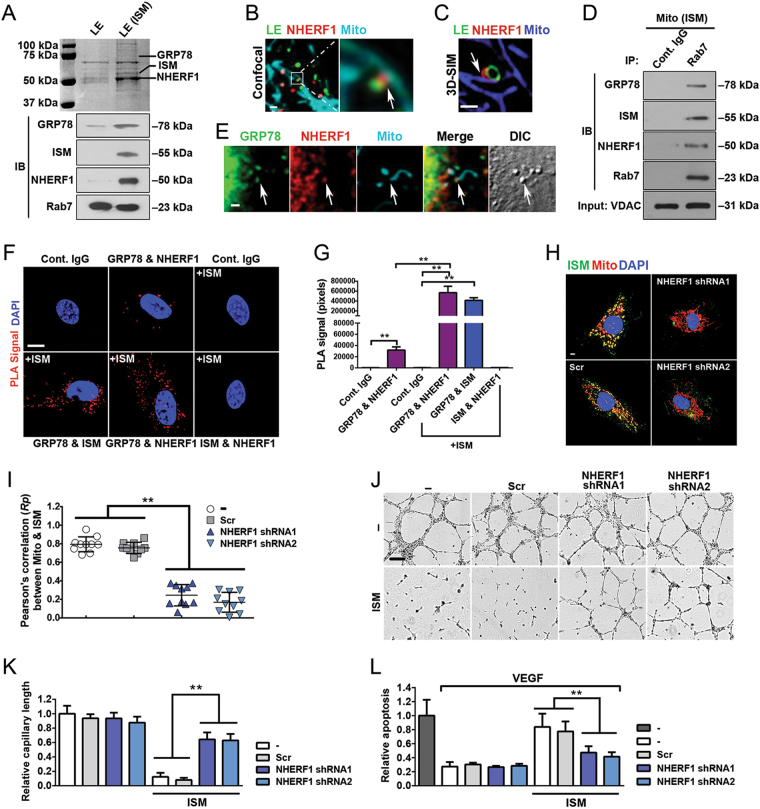
LE-associated NHERF1 plays a role in LE–Mito interaction. **a** Identification of NHERF1 as the most abundant protein in the ISM-treated LE. NHERF1, GRP78, and ISM were identified by MS in the LE fraction from ISM-treated HUVECs and subsequently confirmed by WB. **b** Confocal microscopy revealed that NHERF1 is localized at the interface between LE and Mito. HUVECs labeled by the LE marker (Rab7-GFP) and MitoTracker (cyan) were IF stained for NHERF1 (red). Scale bar, 1 μm. **c** 3D-SIM demonstrated that NHERF1 (red) is localized at the interface between LE (green) and Mito (blue). This frame is a single focal plane. Scale bar, 1 μm. **d** NHERF1 is enriched in the Rab7^+^ Mito fraction of ISM-treated HUVECs. Purified Mito were further pulled down by anti-Rab7 antibody-conjugated beads to isolate LE-interacting Mito. NHERF1, GRP78, and ISM are only present in the Rab7^+^ Mito fraction as determined by WB. VDAC served as the Mito marker and loading control. **e** Confocal imaging showing NHERF1 and GRP78 colocalized in a vesicular structure that was merging with Mito. Scale bar, 1 μm. **f**, **g** PLA demonstrated that NHERF1 and GRP78 directly interacts with each other in the cell, similar to ISM and GRP78. PLA signals (red) indicate two proteins are in close proximity and most likely to directly interact with each other. GRP78-NHERF1 interaction was significantly enhanced by ISM treatment. ISM directly interacts with GRP78 but not NHERF1. Quantification of PLA signal is shown in the bar graph in panel **g**. **P < 0.01, *n* = 10. Error bars denote SD. Scale bar, 5 μm. **h**, **i** Transient transfection of shRNA-expressing constructs targeting NHERF1 effectively blocked the mitochondrial targeting of ISM in HUVECs. A scrambled shRNA construct was transfected as the control, which did not influence the accumulation of ISM in Mito. The colocalization between ISM and Mito was determined by Pearson’s correlation coefficient. **P < 0.01, *n* = 10. Error bars denote SD. Scale bar, 5 μm. **j**, **k** The anti-angiogenic activity of ISM was significantly reduced by shRNA knockdown of NHERF1. ISM treatment suppressed the tube formation of HUVECs on Matrigel. This anti-angiogenic effect was significantly suppressed upon the knockdown of NHERF1. The total tube length was quantified by ImageJ. **P < 0.01, *n* = 3. Error bars denote SD. Scale bar, 200 μm. l The pro-apoptotic function of ISM was suppressed by the knockdown of NHERF1. ISM induced HUVEC apoptosis. This pro-apoptotic functions were significantly reduced upon knockdown of NHERF1. **P < 0.01, *n* = 3. Error bars denote SD

We reasoned that proteins enriched in the endosomes of AS and ISM-treated cells might mediate the interaction between LE and Mito. To identify the enriched proteins in ISM-treated endosomes, we isolated EE and LE fractions and resolved them by sodium dodecyl sulfate-polyacrylamide gel electrophoresis (SDS-PAGE). Enriched protein bands from ISM-treated endosomes were identified by mass spectrometry (MS). Both ISM and its receptor GRP78 were present in EE and LE fractions of ISM-treated HUVECs (Fig. 5a, Fig. S5A). The most enriched protein in ISM-treated EE/LE fractions is Na+/H+ exchanger regulatory factor 1 (NHERF1), also known as ezrin-radixin-moesin-binding protein 50 (EBP50) or solute carrier family 9 isoform 3 regulator 1 (SLC9A3R1). It is a cytoplasmic scaffold protein associated with the PM, known to be involved in regulating the endocytic sorting of β2-adrenergic receptor to prevent it from being sent to lysosomes for degradation [25]. WB confirmed the MS result of NHERF1 enrichment in endosomes upon ISM treatment (Fig. 5a and Fig. S5A). Similarly, AS treatment also enhanced the association of NHERF1 with endosomes, but not FN treatment (Fig. S5, B–E). These results raised the possibility that NHERF1 may play a role in AS and ISM trafficking to Mito. Indeed, confocal and 3D-SIM microscopy indicated that NHERF1 is localized at the interface between the contacting LE and Mito (Fig. 5b, c). We further isolated the fraction of Mito that are interacting with LE by further purifying mitochondrial fraction with anti-Rab7 antibody. NHERF1 level is significantly increased in the Rab7^+^-Mito fraction compared with unfractionated total Mito (Fig. 5d). These findings suggest that LE-associated NHERF1 may facilitate LE–Mito interaction and AS and ISM trafficking to Mito.

As a cytoplasmic protein, NHERF1 functions through interacting with the cytosolic tail of cell surface receptors. Indeed, NHERF1 colocalizes with GRP78, the receptor of ISM, on the cell surface (Fig. S6A). A vesicular structure (likely LE) carrying both the signals of GRP78 and NHERF1 was observed to be converging onto a mitochondrial branch (Fig. 5e). Consistently, GRP78 colocalizes with NHERF1 in LE (Fig. S6B). Proteinase K digestion of purified LE and Mito from ISM-treated HUVECs showed that GRP78 exists in both organelles as a transmembrane protein and in an orientation consistent with its origin and orientation on the cell surface (Fig. S6C) [26, 27]. The N-terminus of GRP78 protrudes into the lumen of LE and Mito and is protected from proteinase K digestion. To verify if NHERF1 can directly associate with GRP78, we performed proximity ligation assay (PLA) and co-immunoprecipitation (co-IP) [28]. As shown in Figs. 5f, g, GRP78 directly interacts with NHERF1 and this interaction is significantly enhanced by ISM treatment. In contrast, no PLA signal was observed between NHERF1 and ISM, indicating no direct interaction between them, consistent with the EE/LE luminal localization of endocytosed ISM. In line with our previous report, ISM directly interacts with GRP78 (Figs. 5f, g) [3]. Co-IP using recombinant NHERF1 and GRP78 confirmed that these two proteins directly interact with each other (Fig. S6D). Knockdown of NHERF1 in HUVECs by short hairpin RNA (shRNA) blocked the translocation of ISM and AS into Mito (Figs. 5h, i; Fig. S7, A-C), and reduced their anti-angiogenic and pro-apoptotic functions (Figs. 5j-l; Fig. S7, D-F). These data demonstrate that NHERF1 interacts with GRP78 on the cytosolic side of LE and facilitates the mitochondrial trafficking, as well as the anti-angiogenic and pro-apoptotic functions of ISM and AS.

### NHERF1 form an interaction complex with SNAP25 to facilitate LE–Mito interaction and mitochondrial trafficking of AS and ISM

**Fig. 6:**
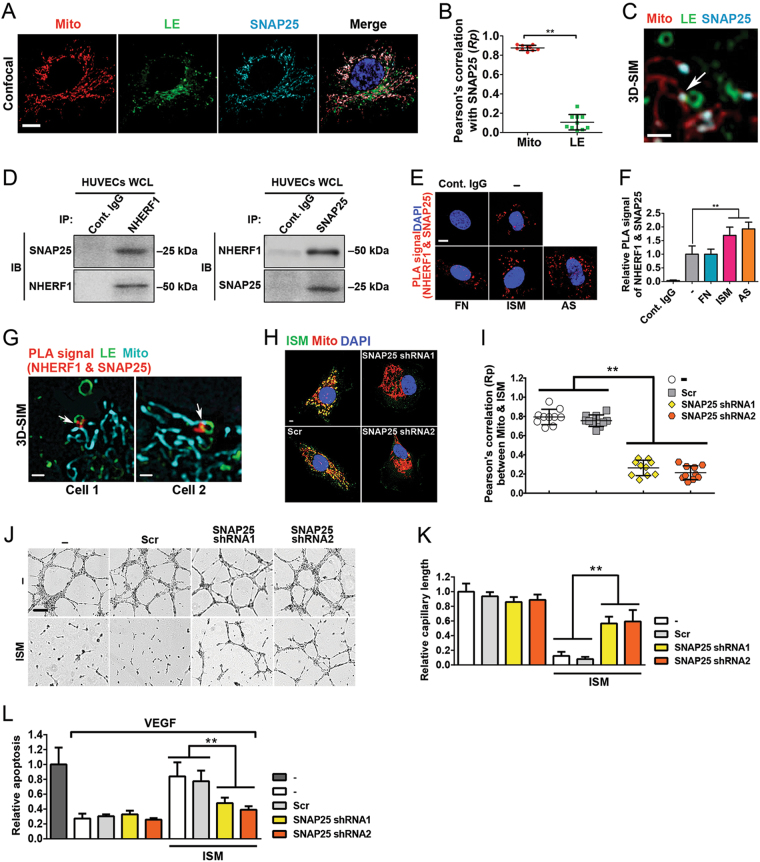
SNAP25 plays a role in the trafficking of AS and ISM to Mito. **a**, **b** SNAP25 is localized in mitochondria in HUVECs. HUVECs labeled by the LE marker (Rab7-GFP) and MitoTracker (Red) was IF stained for SNAP25 (Cyan). SNAP25 is highly colocalized with Mito but not LEs. The colocalization between SNAP25 and Mito was determined by Pearson’s correlation coefficient. **P < 0.01, *n* = 10. Error bars denote SD. Scale bar, 5 μm. **c** SNAP25 is present at the interface between LEs and Mito as determined by 3D-SIM. This frame is a single focal plane. Scale bar, 1 μm. **d** NHERF1 and SNAP25 can co-immunoprecipitate each other from the whole-cell lysate of HUVECs. **e, f** PLA demonstrates that NHERF1 and SNAP25 are localized very close to each other in cells, likely in an interaction complex. AS and ISM treatments further enhance this close localization, but not FN treatment. **P < 0.01, *n* = 10. Error bars denote SD. Scale bar, 5 μm. **g** 3D-SIM super-resolution microscopy demonstrated that NHERF1-SNAP25 interacting complex (PLA signal, red) is localized at the interface of interacting LE (green) and Mito (cyan). HUVECs were labeled by the LE marker (Rab7-GFP) and MitoTracker (cyan) and subjected to PLA (red) analyses. Each frame is a single focal plane. Scale bar, 1 μm. **h, i** Transient transfection of shRNA constructs targeting SNAP25 effectively blocked the mitochondrial targeting of ISM in HUVECs. A scrambled shRNA construct was transfected as the control, which did not influence the accumulation of ISM in Mito. The colocalization between ISM and Mito was determined by Pearson’s correlation coefficient. **P < 0.01, *n* = 10. Error bars denote SD. Scale bar, 5 μm. **j, k** The anti-angiogenic activity of ISM was reduced by the shRNA knockdown of SNAP25. The tube formation of HUVECs on Matrigel was diminished by ISM treatment. This anti-angiogenic effect was significantly suppressed upon the knockdown of SNAP25. The total tube length was quantified by ImageJ. **P < 0.01, *n* = 3. Error bars denote SD. Scale bar, 200 μm. **l** The pro-apoptotic function of ISM was suppressed by the knockdown of SNAP25. ISM induced HUVEC apoptosis. This pro-apoptotic function was significantly reduced upon knockdown of SNAP25. **P < 0.01, *n* = 3. Error bars denote SD

SNARE proteins are the major modulators in intracellular membrane fusion [29]. They are mainly transmembrane proteins, with a few surface-associated members. Synaptosome-associated protein 25  kDa (SNAP25) is a surface-associated target SNARE protein that functions in synaptic vesicle exocytosis in neurons and mediate lipid membrane fusion in a calcium-independent manner [30]. Through literature search, SNAP25 is the only SNARE that was reported to associate with the outer membrane of Mito [31]. As LE–Mito fusion is calcium independent, SNAP25 could be a potential mitochondrial-associated SNARE to participate in LE–Mito interaction. Indeed, SNAP25 is expressed in HUVECs (data not shown). Confocal microscopy revealed that SNAP25 is highly colocalized with Mito but not LE in HUVECs (Figs. 6a, b). By 3D-SIM, intensified SNAP25 signal can be found at the contacting surface between the interacting LE and Mito (Fig. 6c).

Co-IP experiments showed that NHERF1 forms an interaction complex with SNAP25 in HUVECs, with IP of either protein can co-IP the other protein from HUVEC protein lysate (Fig. 6d). Nevertheless, NHERF1 and SNAP25 is unlikely to interact directly with each other as recombinant NHERF1 and SNAP25 failed to co-IP each other (data not shown). PLA results support the notion that NHERF1 and SNAP25 are localized very close to each other in cells and are likely to be in an interaction complex. AS and ISM, but not FN, enhance the formation of NHERF1–SNAP25 interaction complex (Fig. 6e, f). Through 3D-SIM, we found the NHERF1-SNAP25 PLA signal at the contacting surface between LE and Mito (Fig. 6g). Similar to knockdown of NHERF1, knockdown of SNAP25 in HUVECs by shRNA also blocked the translocation of AS and ISM to Mito (Figs. 6h, i, Fig. S8, A-C), and reduced their anti-angiogenic and pro-apoptotic functions (Figs. 6j-l; Fig. S8, D-F).

**Fig. 7:**
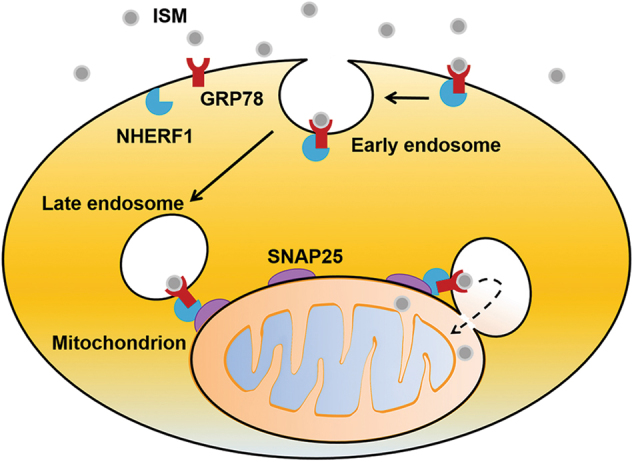
A schematic model for the mitochondrial targeting of extracellular proteins using ISM as an example. Extracellular proteins (ISM) are endocytosed through their cell surface receptors (GRP78). The PM inner surface-associated NHERF1 interacts with the cytoplasmic region of the cell surface receptor (GRP78) and participates in the endosomal sorting of these internalized proteins. LE-associated NHERF1 then interacts with SNAP25 on the mitochondrial surface to pull LE and mitochondria into close proximity. The lipid membranes of LE and Mito interact and merge to facilitate direct protein transfer

Altogether, these results suggest that NHERF1 associated with LE and SNAP25 associated with Mito form an interaction complex to facilitate LE–Mito interaction and/or protein trafficking from LE to Mito (Fig. 7).

## Discussion

In this work, we revealed a novel endosomal protein trafficking pathway that allows extracellular anti-angiogenic proteins such as AS and ISM to be trafficked to Mito through LE. Extracellular AS and ISM make use of this protein trafficking route to reach Mito and execute endothelial cell apoptosis.

Although classic endocytosis destinations include from LE to lysosome and recycling back to PM, other endocytic destinations have recently been reported. For example, endosome was reported to fuse with the nuclear envelope to discharge the endocytosed pseudomonas exotoxin A (PE) into the nucleoplasm [14]. Endosome also interacts with the Golgi apparatus for protein transfer from endosome to Golgi [15]. Our work here adds Mito as another cargo destination for endocytosis. The mitochondrial endosomal trafficking route not only allows extracellular proteins to be trafficked to Mito, it also explains the mitochondrial translocation of PM lipid and cell surface receptors. Incidentally, R18, a membrane-impermeable lipophilic fluorescent dye used as a PM probe, has been reported to translocate into Mito in PC12 neural cells with an unknown mechanism [24].

Mito have been reported to directly interact with the endoplasmic reticulum (ER) [32], Golgi apparatus [33], peroxisomes [34] and melanosome [35]. Endosome and Mito have also been reported to localize at juxtaposition during apoptosis [5]. Meanwhile, direct iron transfer from endosome to Mito have been reported in erythroid cells through a kiss-and-run mechanism [18, 20]. Our work here clearly demonstrate that LE but not EE interacts with Mito and some LEs also merge into Mito. Both membrane fusion and kiss-and-run mechanism can be involved in protein and lipid trafficking from LE to Mito.

We further identified that the cytosolic scaffold protein NHERF1 is enriched on the surface of endosomes upon AS and ISM treatment and plays a critical role in trafficking AS/ISM to Mito. NHERF1 is known to regulate the PM protein levels through intracellular trafficking and recycling [25, 36–38]. It facilitates the recycling of β2-AR to the PM through its PDZ1 domain-mediated interaction and spares β2-AR from heading to lysosome for degradation [25]. It is possible that NHERF1 could also play a role in diverting the endosomal trafficking of AS/ISM from the lysosomal pathway to the Mito pathway. Future studies can investigate this possibility.

NHERF1 is a PDZ domain-containing protein that normally interacts with PDZ ligands. In this work, we discovered that the ISM cell surface receptor, GRP78, is a novel NHERF1-interacting protein. Most PDZ ligands possess a consensus C-terminal amino-acid sequence [39]. NHERF1 interacts with ligands bearing the class I consensus motifs of [Ser/Thr]-X-Φ (X denotes any amino acids and Φ denotes hydrophobic amino acids generally Leu, Ile, Val or Met) [39, 40]. However, more and more evidence has suggested that PDZ proteins may also interact with PDZ ligand proteins through internal sequences within their interacting partners [41–44]. Up to now, no consensus amino-acid sequence from these internal sequences has been revealed. Although GRP78 does not possesses the C-terminal consensus motif required by NHERF1 interaction, it could harbor yet to be identified internal sequence that equips it to be new PDZ ligand.

SNAP25 is a well-characterized SNARE protein participating in the fusion of neurosecretory vesicles at the presynaptic membrane in neurons [30, 31]. However, its function in endothelial cells is not known. Isenmann et al. reported the presence of SNAP25 in endothelial cells and suggested the functional association between SNAP25 and VAMP‐1B in Mito [45]. We show here that SNAP25 is localized at Mito in HUVECs and form an interacting complex with NHERF1 to facilitate LE–Mito interaction or fusion.

The discovery that NHERF1 and SNAP25 are localized at the contacting face between LE and Mito and their interaction complex is induced by AS and ISM suggests that this protein trafficking route is regulated. The cargo protein internalized and its PM receptor may regulate the recruitment of cytosolic protein such as NHERF1 to the endosomal surface. The recruited molecules enriched on the surface of endosomes such as NHERF1 could further determine their subsequent destination.

In summary, this work reveals a previously unrealized endosomal protein trafficking pathway that allows proteins to be trafficked from the extracellular environment to Mito via LE. A schematic diagram of this endosomal trafficking pathway is shown in Fig. 7 using ISM as an example. We demonstrate that LE interacts with and/or merge with Mito, enabling the transfer of not only lipids but also proteins. Extracellular anti-angiogenic proteins, such as AS and ISM, make use of and enhance this endosomal trafficking pathway to reach Mito and execute apoptosis. NHERF1 associated with LE and SNAP25 associated with Mito form an interaction complex that facilitate LE–Mito interaction and protein trafficking to Mito.

## Materials and methods

### Cell culture and reagents

HUVECs were purchased from PromoCell (C-12200, Heidelberg, Germany) and cultured in EndoGRO-LS media (Millipore, MA, USA) as described [46]. EE and LE in HUVECs were labeled by CellLight EE/LE-GFP, BacMam 2.0 for 24 h before fixation or live imaging according to manufacturer’s instructions (Thermo Fisher Scientific). Mito were labeled by MitoTracker Red CMXRos or MitoTracker Deep Red FM (Thermo Fisher Scientific, Rockford, IL, USA) at 200 nM in cell culture medium for 30 min before fixation or live imaging. Recombinant ISM protein and anti-ISM antibody were generated as described [46]. Recombinant AS and FN were from BioVision (Milpitas, CA, USA) and Sigma-Aldrich (St. Louis, MO, USA), respectively. Antibodies for WB, immunofluorescent (IF) staining and co-IP were from Santa Cruz Biotechnology (Santa Cruz, CA, USA) including GRP78 (A10), NHERF1 (H100, A7), SNAP25 (H1), Rab5 (S19), Rab7 (H50), VDAC (D16), LAMP2B (H4B4), TOM20 (F10), AAC (N19), PDI (A1), VE-cadherin (C19), GAPDH (6C5) and β-actin (C4). Antibodies against AS and FN were from Abcam (Cambridge, UK). Antibody for LC3 is from Sigma-Aldrich (L7543). Secondary antibodies were from Thermo Fisher Scientific for IF and Santa Cruz Biotechnology for WB. Clean-Blot IP detection kit (Thermo Fisher Scientific) was used for co-IP. The endosome-destabilizing peptide L17E (IWLTALKFLGKHAAKHEAKQQLSKL-CONH2) [17] was synthesized by Genscript (Piscataway, NJ, USA). Recombinant VEGF_165_ was purchased from R&D system (Minneapolis, MN, USA). Other chemicals were from Sigma-Aldrich.

### IF staining

HUVECs were seeded onto glass coverslips coated with 0.2% gelatin. EE (Rab5-GFP baculovirus)/LE (Rab7-GFP baculovirus) labeled or MitoTracker dye-labeled cells were fixed with 4% paraformaldehyde (PFA) in phosphate-buffered saline (PBS) for 30 min at room temperature and permeabilized with 0.3% Triton in PBS for 15 min. After blocking with 3% bovine serum albumin (BSA) in PBS for 2 h, cells were incubated with primary antibody of appropriate dilutions for 1 h. After washing 3× by PBS with 0.1% Tween20 (PBST), cells were stained with corresponding secondary antibody. Cells were again washed 3× with PBST before been mounted with Fluoroshield mounting buffer with 4,6-diamidino-2-phenylindole (DAPI).

### Confocal microscopy

Images of fixed cells were collected by Zeiss LSM-510 Meta confocal microscope (Carl Zeiss AG, Oberkochen, Germany) or the UltraView VoX spinning disk confocal microscope (Perkin Elmer, Waltham, MA, USA). The colocalization (Pearson’s correlation coefficient, Rp) was analyzed by Imaris software (Bitplane Inc., South Windsor, CT, USA). For live-cell imaging, HUVECs were cultured in glass-bottomed µ-Dish of 35 mm (ibidi GmbH, Martinsried, Germany) coated with 0.2% gelatin. Time-lapse imaging was conducted by the UltraView VoX spinning disk confocal microscope at 37°C with 5% CO_2_. Images were collected every 15 s for 11 min at the maximum speed.

### 3D structured illumination microscopy

HUVECs were seeded on high-precision cover glasses of thickness no. 1.5H (Deckgläser, Glaswarenfabrik Karl Hecht, Sondheim, Germany) for super-resolution microscopy, processed as indicated and mounted with VectaShield H-1000 mounting medium (Vector Laboratories, Burlingame, CA, USA). A DeltaVision OMX v4 Blaze microscope (GE Healthcare, Piscataway, NJ, USA) equipped with 405, 488, 568 and 642 nm lasers for excitation and the BGR-FR filter drawer (emission wavelengths 436/31 for DAPI, 528/48 for Rab5/7-GFP, 609/37 for MitoTracker Red CMXRos or Alexa568, and 683/40 for MitoTracker Deep Red) was used for the acquisition of 3D-SIM images. An Olympus Plan Apochromat 100×/1.4 PSF oil immersion objective lens was used (1.514 refractive index immersion oil) with multiple liquid-cooled Photometrics Evolve EM-CCD cameras. Fifteen images per section per channel were acquired (made up of three rotations and five phase movements of the structured illumination pattern) at a z-spacing of 0.125 µm as previously described [47, 48]. Structured illumination reconstruction and channel alignment were completed using the SoftWorX program (GE Healthcare). The 3D-SIM images were 3D-rendered by Imaris x64 8.4.1 (Bitplane Software). To show the full membrane structure of interacting LE and Mito, the z-range for cell 1 is 750 nm, for cell 2 is 600 nm, for cell 3 is 1125 nm. Scale bar, 0.5 μm.

### Isolation of endosomes and Mito

EE and LE of HUVECs were isolated through immunoisolation or sucrose step-gradient centrifugation [49, 50]. After 3 h of treatment by AS (500 nM), ISM (1 μM) or FN (10 μg/ml), HUVECs were scraped down into ice-cold hypotonic buffer containing 10 mM Tris-HCl of pH 7.9, 0.5 mM DTT and 1× protease inhibitor cocktail solution (Roche). Cells were homogenized in a tight-fitting Dounce homogenizer (Wheaton, Millville, NJ, USA) with 50 strokes. The homogenate was centrifuged at 8000 *g* for 10 min at 4°C to remove the nuclei and Mito. For immunoisolation method, protein A/G agarose beads pre-bound with anti-Rab5 (EE marker)/anti-Rab7 (LE marker) antibodies were used to pull down EE/LE in the supernatant, respectively [49]. For sucrose step-gradient centrifugation method, the supernatant is then subjected to sucrose step density gradient centrifugation as described [50]. Mito were isolated using the Mito isolation kit for cultured cells (Thermo Fisher Scientific) or sucrose step-gradient centrifugation [51]. To obtain the hybrid structure of interacting LE–Mito, purified Mito from HUVECs were further pulled down using anti-Rab7 antibody.

### Labeling of PM

The PM of HUVECs was labeled by the lipophilic cell tracking dye R18 (Thermo Fisher Scientific), PKH26 (Sigma-Aldrich) and CellVue® Maroon (Polysciences, Warrington, PA, USA). For R18 labeling, HUVECs were trypsinized and resuspended in PBS containing 20 μM R18. After 10-min incubation at 37 °C, the excessive R18 dye was neutralized by an equal volume of 1% BSA in PBS for 1 min. Cells were then washed 3× with complete medium and processed for subsequent analysis. PKH26 labeling of the PM was done using the PKH26 red fluorescent cell linker kit. For labeling of the PM with the far-red dye, the CellVue® Maroon Kits for Membrane Labeling were used according to the manufacturer’s instruction. MitoTracker Deep Red FM was added 45 min before fixation for imaging. To check the lipid transfer from the PM into Mito, HUVECs labeled by R18/PKH26 were treated by AS, ISM or FN for 3 h. Mitochondrial fraction was then isolated and measured by Infinite 200 PRO fluorometric reader (Tecan, Männedorf, Switzerland) at 551/567 nm for PKH26 and 560/610 nm for R18.

### FRAP analysis of the mitochondrial targeting of LEs

HUVECs double labeled by LE (Green, Rab7-GFP-expressing baculovirus) and Mito (Red, MitoTracker) were treated with AS (500 nM), ISM (1 μM) or FN (10 μg/ml) for 3 h before FRAP experiment using an UltraView VoX spinning disk confocal microscope (Perkin Elmer). A defined area filled with Mito was chosen as the region of interest (ROI; 4.5 × 4.5 *μ*m). The initial GFP signal of LE in ROI was recorded every 15 s for 1 min. Following the photo-bleaching of this area by full laser power for 1 s, the fluorescence recovery of GFP signal was monitored by scanning the ROI at low laser power every 15 s for 10 min at the maximum speed, and subsequently analyzed. FRAP recovery curves denoting the mobility of LEs (T_1/2_) were generated by Volocity (Perkin Elmer).

### Protein analyses from LE and Mito by MS and WB

EEs or LEs were isolated from HUVECs with or without 3-h treatment of ISM. An equal amount of endosomal lysates from ISM-treated HUVECs and untreated cells was separated by SDS-PAGE. Protein bands were visualized by InstantBlue staining (Expedeon, San Diego, CA, USA) for 15 min. Specific or enriched bands in the endosomal lysates from ISM-treated HUVECs were analyzed by MALDI-TOF-TOF MS (AB SCIEX, Framingham, MA, USA). The results were searched against NCBInr 080723 database. All the MS-identified proteins were confirmed by WB. The existence of the identified protein in the endosomal fractions of AS and FN-treated cells was also checked by WB.

### Proximity ligation assay

HUVECs treated by AS (500 nM), ISM (1 μM) or FN (10 μg/ml) for 3 h as indicated were fixed by 4% PFA and processed for PLA. The direct interactions between NHERF1 and GRP78, GRP78 and ISM, NHERF1 and ISM, NHERF1 and SNAP25 were determined by the red PLA signals from Duolink in situ red starter kit mouse/rabbit (Sigma-Aldrich). The PLA signal between normal mouse and normal rabbit IgG (Santa Cruz Biotechnology) were recorded as the Control.

### HUVECs tube formation assay

The HUVEC tube formation assay was performed in a μ-Slide Angiogenesis chamber (ibidi GmbH) using in vitro angiogenesis assay kit (Millipore) following the manufacturer’s instruction. HUVECs (1 × 10^4^) were seeded into each well and treated with AS (500 nM), ISM (1 μM), FN (10 μg/ml) and the endosome-destabilizing peptide L17E (40 µM) in 50 μl of EndoGRO basal media (Millipore) containing 2% fetal bovine serum (FBS) for 6 h before imaging. To study the role of NHERF1/SNAP25 in the anti-angiogenic function of AS/ISM, HUVECs were transfected with shRNA and 48 h later were treated as indicated. The images were recorded by Axio Imager M2 (Carl Zeiss AG) and the total tubular length was quantified by ImageJ.

### Apoptosis assay

HUVECs (2 × 10^4^) were seeded into 96-well plate and starved in EndoGRO basal media with 2% FBS for 3 h before they were treated for 24 h with AS (500 nM), ISM (1 μM), FN (10 μg/ml) and the endosome-destabilizing peptide L17E (40 µM) in 50 μl of EndoGRO basal media with 2% FBS and 15 ng/ml VEGF (R&D systems). To check the role of NHERF1/SNAP25 in the pro-apoptotic function of AS/ISM, HUVECs at 48 h post shRNA transient transfection were used and treated as indicated. Apoptosis was determined by a DNA fragmentation cell death ELISA kit (Roche, Basel, Switzerland).

### Lipid-mixing assay

The R18-labeled LE (LE-R18) and Mito (Mito-R18) were isolated from HUVECs treated with 20 μM R18 dye. The unlabeled LE and Mito were isolated from HUVECs without R18 dye treatment. A high-salt wash using 2 M NaCl (S) and administration of 1 mM Ca^2+^ or 2 mM EGTA were applied to study whether the interaction is mediated by surface-associated proteins or in a calcium-dependent manner [52]. Equal amounts of LE and Mito-R18 or LE-R18 and Mito were mixed in 96-well plate in PBS. The kinetics of fluorescence at 560 nm/610 nm was monitored for 90 min at 37 °C by Hidex sense microplate reader (LabLogic, Brandon, FL, USA). The fluorescent signal in each well was read every 10 min for 10 cycles. The kinetic curve (the percentage of fluorescent change) was calculated according to (F(n)/F(1)–1) × 100. Each group has triplicate wells and the experiment was repeated 3×.

### Total internal reflection fluorescence microscopy

The R18-labeled LEs (LE-R18) were isolated from HUVECs treated with 20 μM R18 dye. The MitoTracker Green-labeled Mito were isolated from HUVECs without R18 treatment. Purified LE (Red, R18 lipid dye labeled) and Mito (labeled by MitoTracker Green) were mixed in PBS in a poly-d-lysine (PDL)-coated Petri dish. Their direct interaction was examined by TIRFM performed using a Nikon Ti inverted microscope with three laser lines (491, 561 and 642 nm). The microscope was equipped with an iLAS2 motorized TIRF illuminator (Roper Scientific GmbH) and with a Prime 95B sCMOS camera (Photometrics, 16 bit, pixel size 11 μm). All TIRF images were acquired using Nikon objectives (Apo TIRF 100×, N.A. 1.49 oil). Samples were imaged in two channels by sequential excitation with the laser at 491 and 561 nm through a quad-bandpass filter (Di01-R405/488/561/635, Semrock, Rochester, NY, USA) for both channels, a 520/35 filter (Semrock) for 491 nm channel, and a 641/75 filter (Semrock) for 561 nm channel. All images were acquired at 1 Hz. The microscope was controlled by Metamorph 7.10 software (Universal Imaging, Bedford Hills, NY, USA). Time-lapse images were taken per second for 10 min. All images were acquired at 37 °C throughout the experiment. The kinetic curve of the fluorescent intensity of the red (R18) and green (MitoTracker Green) channels was generated by ImageJ.

### Proteinase K digestion

Mitochondria/LEs were isolated from HUVECs post 24/3-h treatment of 1 μM ISM or control cells as described above. The isolated mitochondria/LEs were subjected to incubation with 200 μg/ml proteinase K (Roche) or just PBS for 30 min at 4 °C. The proteolytic cleavage reactions were terminated by the addition of 500 μM phenylmethylsulfonyl fluoride (PMSF). Digested mitochondria/LEs were lysed in SDS-PAGE loading buffer at 100°C for 5 min and analyzed by WB.

### RNAi (RNA interference)

The shRNA targeting sequence was designed by BLOK-iT RNAi designer (Invitrogen) or referencing to literature. For silencing human NHERF1, two shRNA constructs in pRFP-C-RS (OriGene Technologies, Rockville, MD, USA) targeting 5ʹ-GGAAACTGACGAGTTCTTCAAGAAATGCA-3ʹ and 5ʹ-CCCATCCTAGACTTCAACA-3ʹ were built. To knockdown human SNAP25, two shRNA constructs in pRFP-C-RS targeting 5ʹ-GTAACAAGCTTAAATCAAGTGATGCTTAC-3ʹ and 5ʹ-GGTTGTACATAGTGGTCATTT-3ʹ were built. A non-effective scramble shRNA cassette (5ʹ-GCACTACCAGAGCTAACTCTTATAGTCCT-3ʹ) in pRFP-C-RS was used as a RNAi control vector. HUVECs (5 × 10^5^) were transfected with 2 μg of shRNA construct by Nucleofector II (Amaxa Biosystems, Lonza, Basel, Switzerland) under program A034. After 48 h, HUVECs were treated by AS (500 nM) or ISM (1 μM) for 3 h and then incubated with MitoTracker Deep Red FM (200 nM) for 30 min. The mitochondrial targeting of AS and ISM upon NHERF1 knockdown was visualized by IF.

### Statistical analysis

Data were expressed as the mean ± standard deviation (±SD). Statistical significance was determined using one-way analysis of variance (ANOVA). *P < 0.05; **P < 0.01, *n* > 3.

## Electronic supplementary material

Figure S1

Figure S2

Figure S3

Figure S4

Figure S5

Figure S6

Figure S7

Figure S8

Supplementary Information

Video 1

Video 2

Video 3
