# Supplementary figures and images for: Extracellular anti-angiogenic proteins augment an endosomal protein trafficking pathway to reach mitochondria and execute apoptosis in HUVECs
Source: Cell Death Differ. 2018 Mar 9;25(11):1905–20. doi: 10.1038/s41418-018-0092-9 (PMC6219483; doi:10.1038/s41418-018-0092-9)

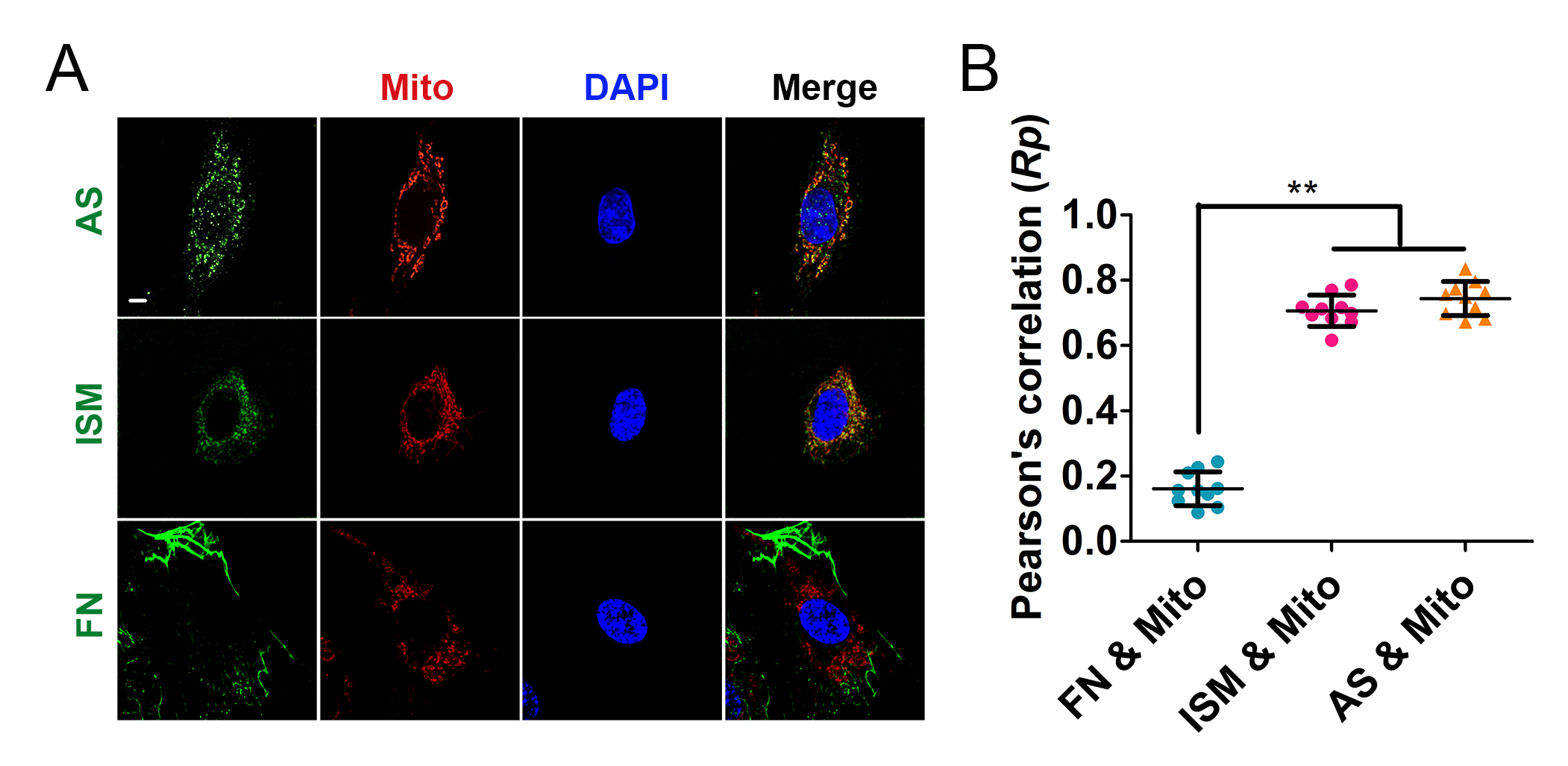

Supplement: Supplementary file 1 — Figure S1 [file 41418_2018_92_MOESM1_ESM.jpg]

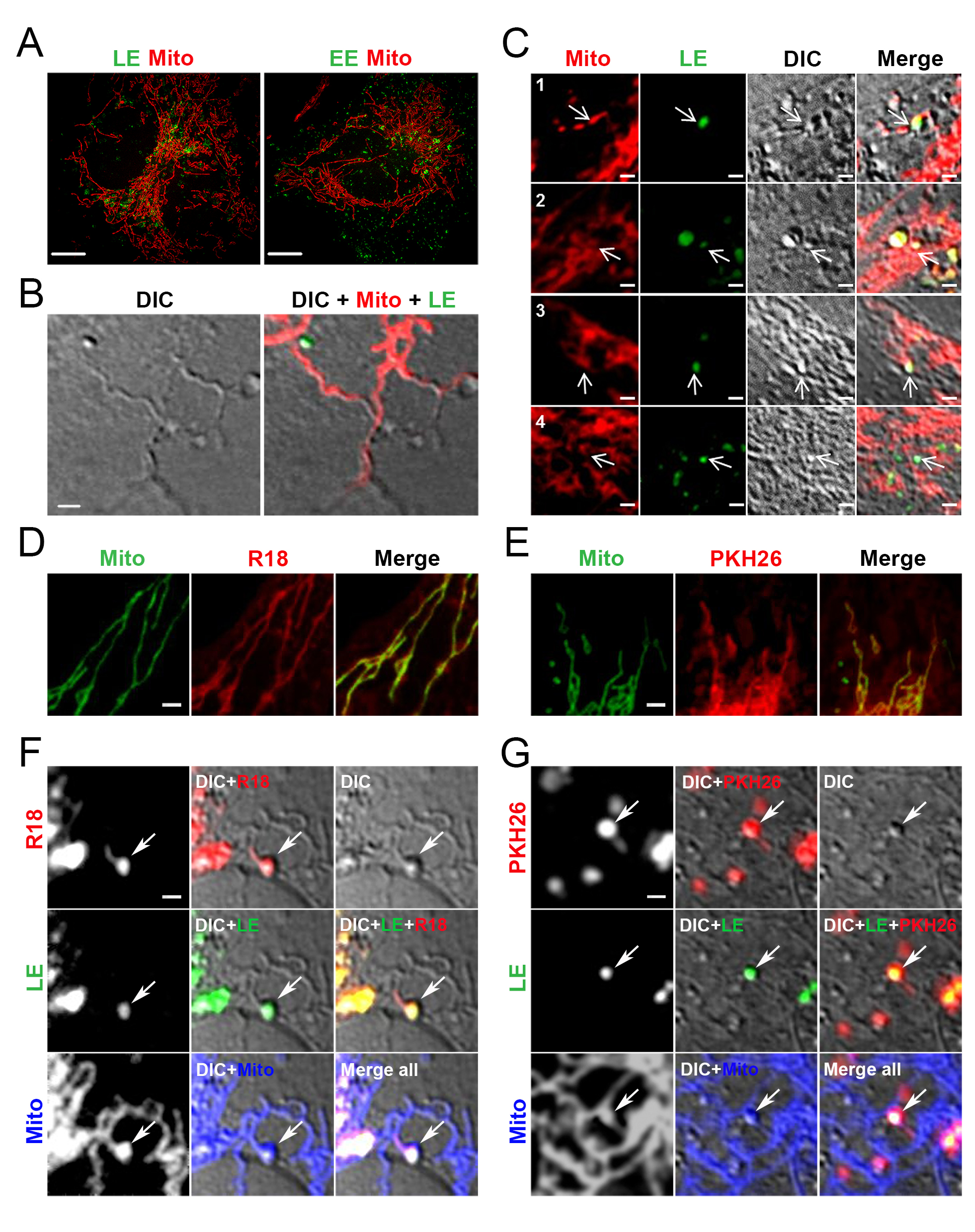

Supplement: Supplementary file 2 — Figure S2 [file 41418_2018_92_MOESM2_ESM.jpg]

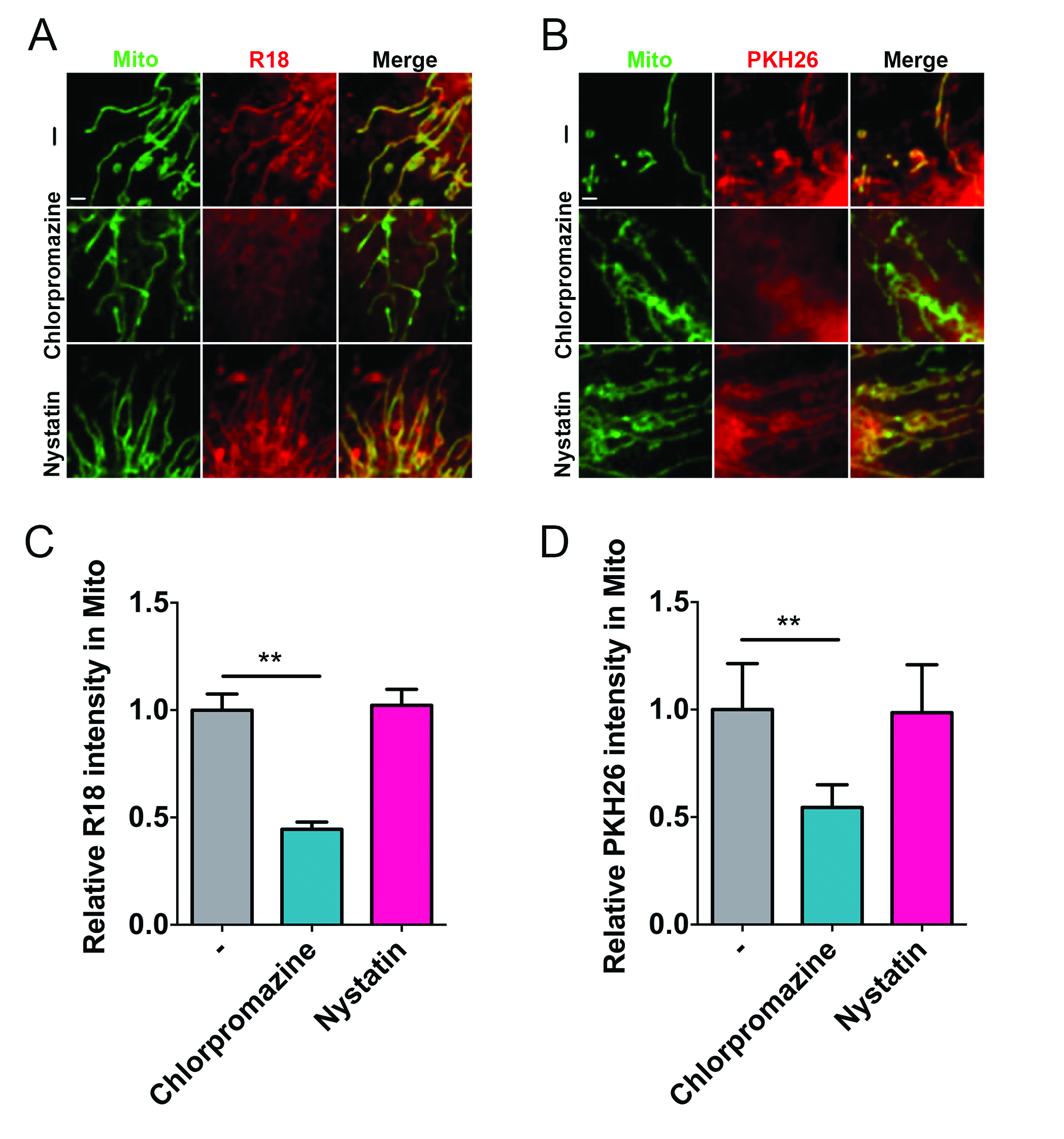

Supplement: Supplementary file 3 — Figure S3 [file 41418_2018_92_MOESM3_ESM.jpg]

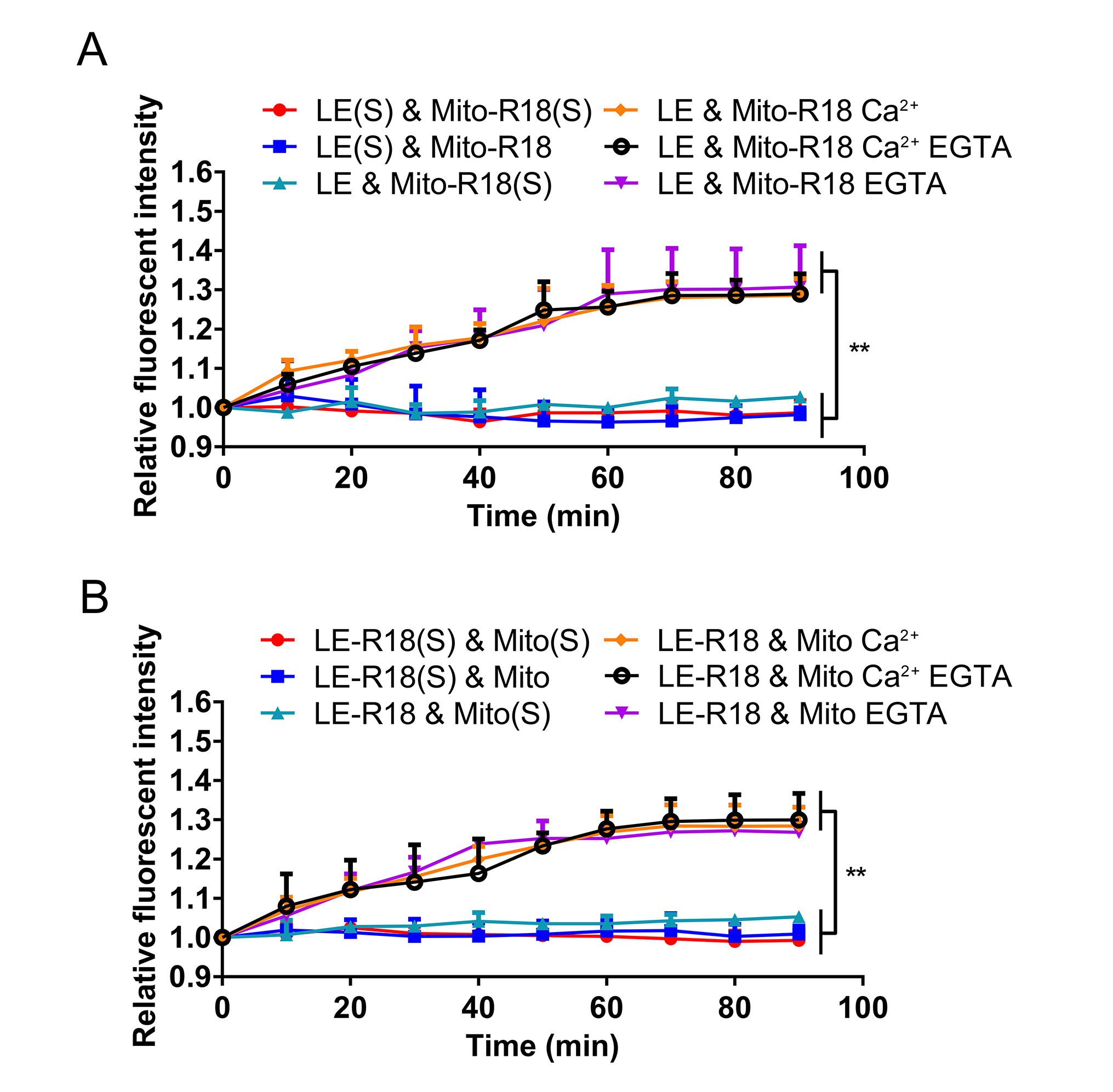

Supplement: Supplementary file 4 — Figure S4 [file 41418_2018_92_MOESM4_ESM.jpg]

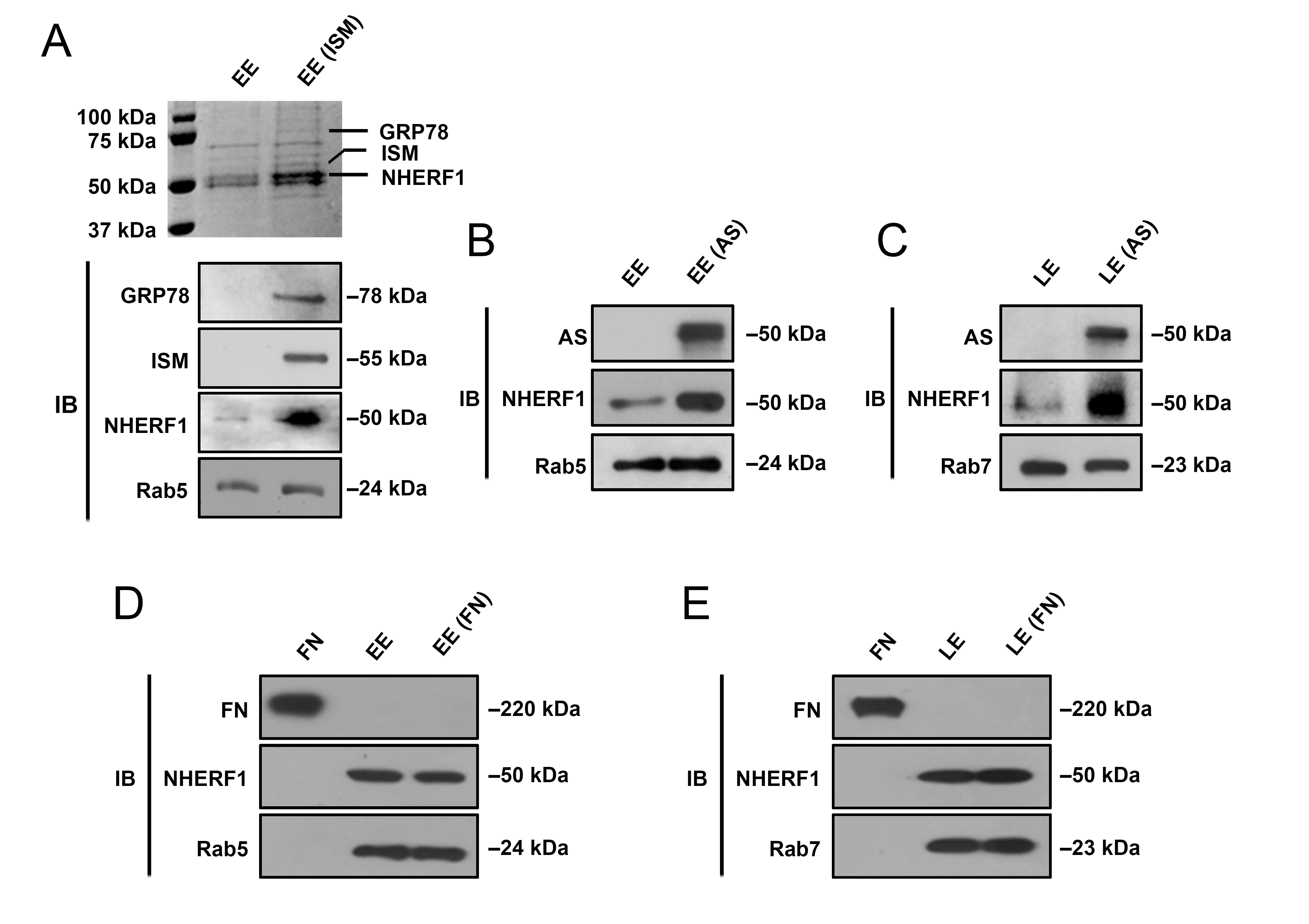

Supplement: Supplementary file 5 — Figure S5 [file 41418_2018_92_MOESM5_ESM.jpg]

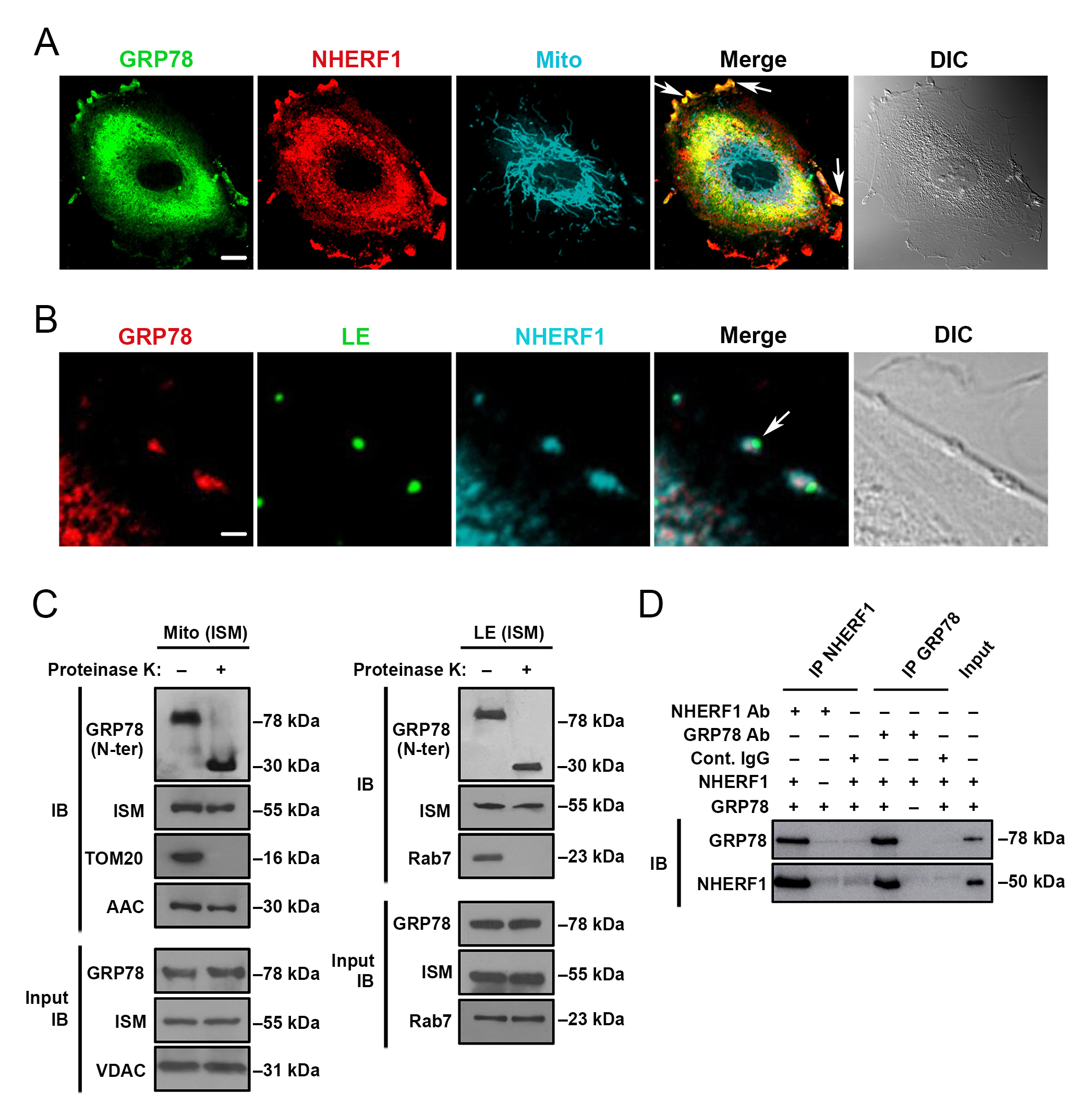

Supplement: Supplementary file 6 — Figure S6 [file 41418_2018_92_MOESM6_ESM.jpg]

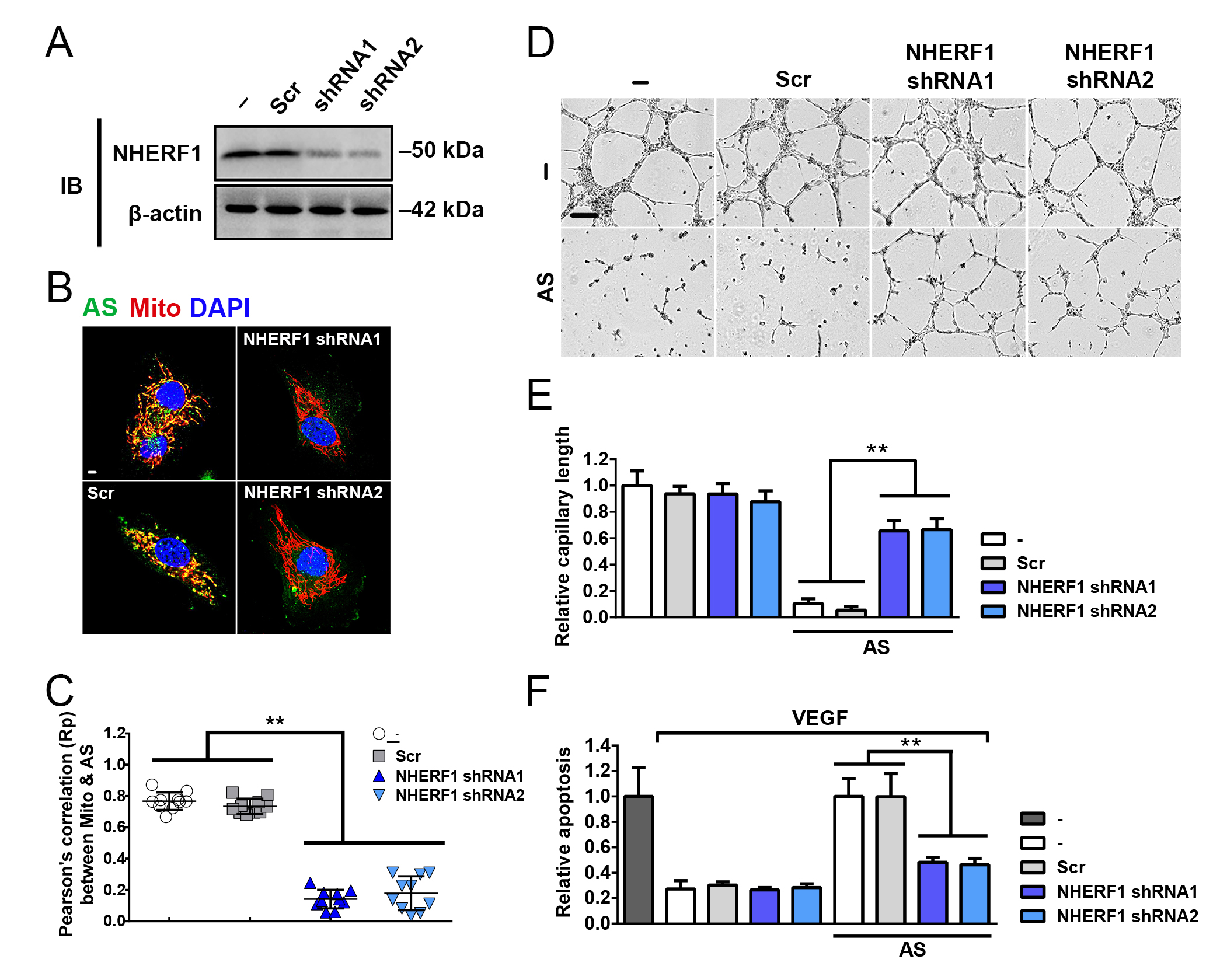

Supplement: Supplementary file 7 — Figure S7 [file 41418_2018_92_MOESM7_ESM.jpg]

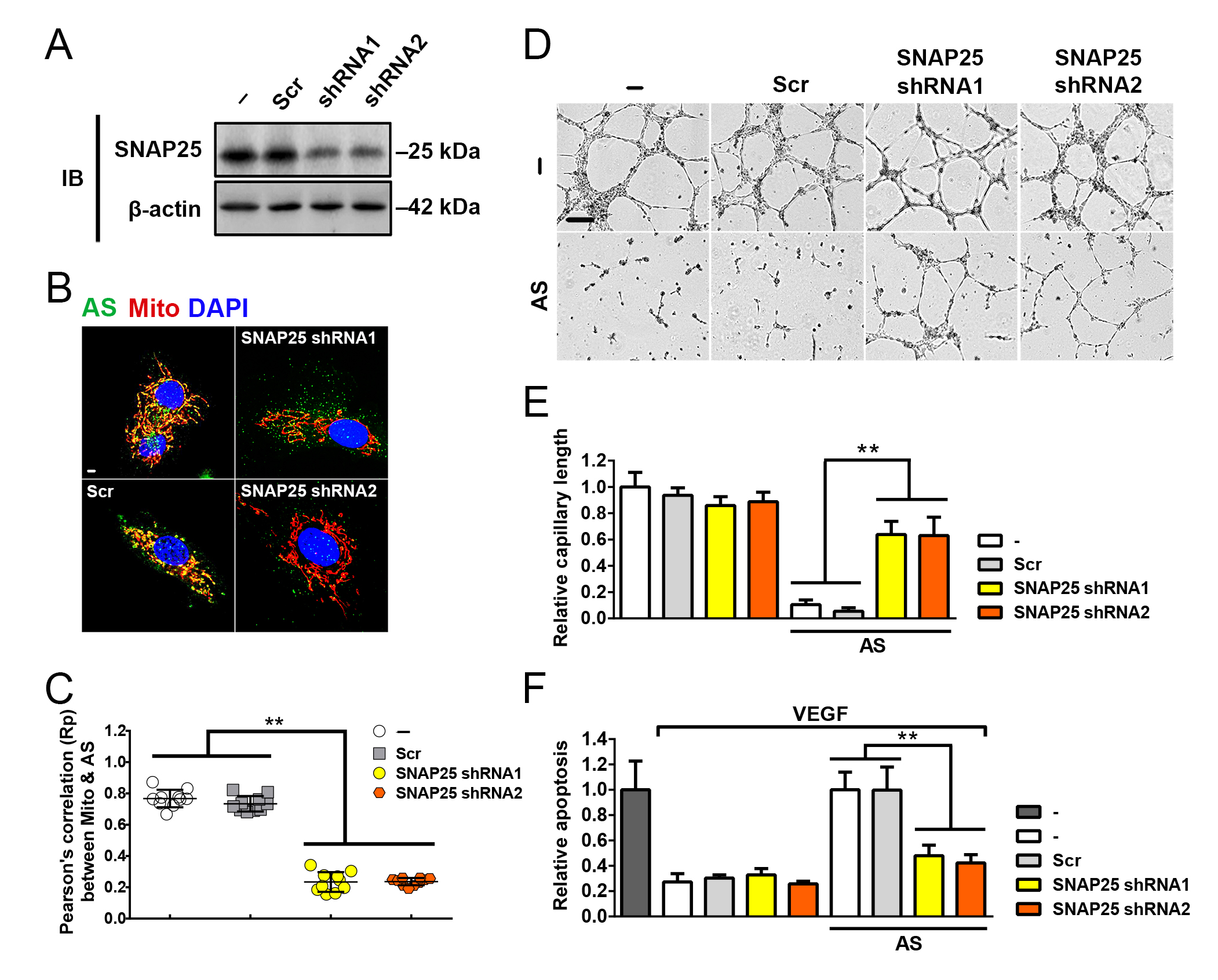

Supplement: Supplementary file 8 — Figure S8 [file 41418_2018_92_MOESM8_ESM.jpg]
