# Supplementary material for: Extracellular anti-angiogenic proteins augment an endosomal protein trafficking pathway to reach mitochondria and execute apoptosis in HUVECs
Source: Cell Death Differ. 2018 Mar 9;25(11):1905–20. doi: 10.1038/s41418-018-0092-9 (PMC6219483; doi:10.1038/s41418-018-0092-9)
Supplement: Supplementary file 9 — Supplementary Information [file 41418_2018_92_MOESM9_ESM.docx]

**Supplementary Information**

**Figure S1. Internalized AS and ISM are targeted to mitochondria.** (**A**) Extracellular AS/ISM were internalized into HUVEC and targeted to mitochondria. FN, which does not target to mitochondria, served as a control. HUVECs treated by AS/ISM/FN for 3 h were labelled with MitoTracker (Red) and IF stained for AS/ISM/FN. Scale bar, 5 μm. (**B**) The co-localization between AS/ISM/FN and Mito was determined by Pearson’s correlation coefficient. ***P*<0.01, n=10.

**Figure S2. LEs directly interact with Mito and engage in lipid transfer.** (**A**) Subcellular distribution patterns of EE/LE and Mito determined by 3D-SIM. HUVECs were labelled by the EE/LE marker (Rab5-GFP/Rab7-GFP) and MitoTracker (Red). Shown here is a full maximum-intensity projection along the whole z of cell depth that contains all the branchial mitochondria and vesicular endosome. Scale bar, 1 μm. (**B**) The vesicular LE and the branching Mito can be clearly resolved under DIC. HUVECs were labelled by LE marker (Rab7-GFP) and MitoTracker (Far-red). Scale bar, 1 μm. (**C**) Interacting LE and Mito merged into a single structure under DIC. Panel 1-3: interacting LE and Mito merged into a single structure in a seamless transition under DIC (arrow). The protein contents of Mito (MitoTracker) and LE merged. Panel 4: LE and Mito located at juxtaposition displayed as two distinct structures with a clear gap in-between (arrow). Scale bar, 1 μm. (**D, E**) The R18/PKH26 dye used to label the PM of HUVECs showed a clear distribution in mitochondria. Scale bar, 1 μm. (**F, G**) The R18/PKH26 lipid dye from the PM transferred from LE into Mito. The R18/PKH26 lipid dye (red) was used to label the PM of HUVECs labelled by LE marker (Rab7-GFP) and MitoTracker (blue). HUVECs were processed immediately for confocal microscopy after R18/PKH26 staining. Scale bar, 1 μm.

**Figure S3. Clathrin-dependent endocytosis contributes to the lipid transfer from plasma membrane to the mitochondria in HUVECs.** (**A, B**) Inhibition of clathrin-dependent endocytosis reduced the transfer of R18/PKH26 labeled lipid from plasma membrane to mitochondria. Chlorpromazine: inhibitor of clathrin-dependent endocytosis, 3 μg/ml Chlorpromazine; Nystatin: inhibitor of clathrin-independent endocytosis, 25 μg/ml Nystatin. Representative images of mitochondria from confocal fluorescent microscopy are shown. Mitochondria are labelled with MitoTracker green and R18/PKH26 are in red. Scale bars, 1 μm. (**C, D**) Translocation of R18/PKH26 labelled lipid dye from plasma membrane to mitochondria was partially blocked by chlorpromazine, but not by nystatin. Quantifications of R18/PKH labelled lipid translocated from PM to Mito were achieved through quantification of fluorescent signals of purified mitochondrial fractions. **P<0.01, n=3. Error bars denote SD.

**Figure S4. The interaction between LEs and Mito is mediated by LE/Mito surface-associated proteins and in a calcium-independent manner.** (**A**) The kinetics of fluorescence de-quenching in the *in vitro* lipid-mixing assay using non-labelled LEs and R18-labelled mitochondria (Mito-R18). This interaction was blocked by a high salt wash (S) of either LEs or Mito and was not affected by the administration of EGTA (2 mM) or Ca^2+^ (1 mM). ***P*<0.01, n=3. Error bars denote SD. (**B**) The kinetics of fluorescence de-quenching of the *in vitro* lipid-mixing assay between R18-labelled LEs (LE-R18) and non-labelled Mito. This interaction was also blocked by a high salt wash (S) of either LEs or Mito and was not affected by the administration of EGTA or Ca^2+^. ***P*<0.01, n=3. Error bars denote SD.

**Figure S5. NHERF1 is highly enriched in the endosomal fraction of AS and ISM treated HUVECs.** (A) NHERF1 is enriched in the EEs of ISM-treated HUVECs. (B) NHERF1 is enriched in the EEs of AS-treated HUVECs. (C) NHERF1 is enriched in the LEs of HUVECs treated by AS. (D) FN, which does not target to mitochondria, does not induce the enrichment of NHERF1 in EEs. (E) FN does not induce the enrichment of NHERF1 in LEs.

**Figure S6.** **GRP78 is co-localized with NHERF1 and directly interacts with NHERF1.** (**A**) Confocal imaging showing NHERF1 and GRP78 are co-localized on the cell surface, cytosol and some Mito region. Scale bar, 5 μm. (**B**) NHERF1 co-localizes with GRP78 in the LEs. The overlapped IF signals of NHERF1 and GRP78 were observed in LEs, suggesting that NHERF1 and GRP78 are associated during endocytosis. HUVECs labelled by the LE marker (Rab7-GFP) were double stained with antibodies against NHERF1 (Cyan) and GRP78 (Red). The cellular structure was observed by DIC. Scale bar, 1 μm. (**C**) GRP78 exists as a transmembrane protein on the outer membrane of mitochondria and the membrane of LEs. Isolated Mito or LEs from HUVECs were subjected to proteinase K digestion or incubated under the same condition without proteinase K. At the end of the reaction, the amount of GRP78 was detected by WB using the mouse monoclonal anti-GRP78 antibody recognizing the N-terminus. Digested GRP78 fragment around 30 kDa was detected. TOM20 and AAC were used as the control for mitochondrial outer membrane and inner membrane protein respectively. VDAC was used as the input control for mitochondria. Rab7 was used as the input control for LEs. In both Mitoand LEs, GRP78 exists as a transmembrane protein with its N-terminus protruding into the lumen of both organelles. ISM is localized inside the mitochondria and LEs and is protected from proteinase K digestion. (**D**) NHERF1 directly interacts with GRP78 as demonstrated by co-IP with recombinant proteins. Lane 1, 2, 3: GRP78 was co-immunoprecipitated by anti-NHERF1 antibody; Lane 4, 5, 6: NHERF1 was co-immunoprecipitated by anti-GRP78 antibody; Lane 7: 1% of the pure protein used in co-IP. Control IgG was from the same species of the pull-down antibodies.

**Figure S7. Knockdown of NHERF1 by shRNA blocked the mitochondrial targeting of AS.** (**A**) WB confirmed the effective NHERF1 knockdown in HUVECs by shRNA constructs. (**B, C**) Transient transfection of shRNA expressing constructs targeting NHERF1 effectively blocked the mitochondrial targeting of AS in HUVECs. A scrambled shRNA construct was transfected as the control, which did not influence the accumulation of AS in Mito. The co-localization between AS and Mito was determined by Pearson’s correlation coefficient. ***P*<0.01, n=10. Error bars denote SD. Scale bar, 5 μm. (**D, E**) The anti-angiogenic activity of AS was reduced by the shRNA knockdown of NHERF1. The total tube length was quantified by ImageJ. ***P*<0.01, n=3. Error bars denote SD. Scale bar, 200 μm. (**F**) The pro-apoptotic function of AS was suppressed by the knockdown of NHERF1. ***P*<0.01, n=3. Error bars denote SD.

**Figure S8.** **Knockdown of SNAP25 by shRNA blocked the mitochondrial targeting of AS.** (**A**) WB confirmed the effective SNAP25 knockdown in HUVECs by shRNA constructs. (**B, C**) Transient transfection of shRNA expressing constructs targeting SNAP25 effectively blocked the mitochondrial targeting of AS in HUVECs. A scrambled shRNA construct was transfected as the control, which did not influence the accumulation of AS in Mito. The co-localization between AS and Mito was determined by Pearson’s correlation coefficient. ***P*<0.01, n=10. Error bars denote SD. Scale bar, 5 μm. (**D, E**) The anti-angiogenic activity of AS was reduced by the shRNA knockdown of SNAP25. The total tube length was quantified by ImageJ. ***P*<0.01, n=3. Error bars denote SD. Scale bar, 200 μm. (**F**) The pro-apoptotic function of AS was suppressed by the knockdown of SNAP25. ***P*<0.01, n=3. Error bars denote SD.

**Video S1. Kiss-and-run phenomenon between LE and Mito.**

**Video S2. LE-Mito interaction.**

**Video 3. LE-Mito interaction.**

**Supplementary Method:**

**Effect of endocytosis inhibition on lipid transfer from plasma membrane to mitochondria**

To check the contribution of endocytosis to lipid transfer from plasma membrane to mitochondria, HUVECs were pre-treated by 3 μg/ml chlorpromazine (inhibitor of clathrin-dependent endocytosis) or 25 μg/ml nystatin (inhibitor of clathrin-independent endocytosis) for 30 min and then labeled with R18 or PKH26 lipid dye in the presence of these inhibitors. Cells were then seeded onto glass coverslips and continuously treated with the inhibitors in full media for 3 h. MitoTracker was added at 45 min before fixation. Cells were fixed and processed for imaging. To quantify the lipid transfer from plasma membrane into mitochondria, HUVECs labeled by R18 or PKH26 were treated by 3 μg/ml chlorpromazine or 25 μg/ml nystatin in full media for 3 h. Mitochondrial fractions were then isolated from these cells and their R18/PKH26 fluorescent signals were measured by Infinite 200 PRO fluorometric reader (Tecan).
